# Bioengineering the Junctional Epithelium in 3D Oral Mucosa Models

**DOI:** 10.3390/jfb15110330

**Published:** 2024-11-06

**Authors:** Marianna Gavriiloglou, Mira Hammad, Jordan M. Iliopoulos, Pierre Layrolle, Danae A. Apazidou

**Affiliations:** 1 Department of Preventive Dentistry, Periodontology & Implant Biology, School of Dentistry, Aristotle University of Thessaloniki, GR-54124 Thessaloniki, Greece; mariangavril@outlook.com (M.G.); perioapatzidou@yahoo.gr (D.A.A.); 2 Toulouse NeuroImaging Center (ToNIC), INSERM, Toulouse University UMR 1214, CHU Toulouse Purpan, 31024 Toulouse, France; mira.hammad@inserm.fr; 3 Faculty of Health Sciences, School of Medicine, Aristotle University of Thessaloniki, GR-54124 Thessaloniki, Greece; jordaniliopoulos@yahoo.gr

**Keywords:** 3D oral mucosa model, gingiva, junctional epithelium, implant insert, tooth insert, soft-tissue barrier, fibroblasts, keratinocytes

## Abstract

Two-dimensional (2D) culture models and animal experiments have been widely used to study the pathogenesis of periodontal and peri-implant diseases and to test new treatment approaches. However, neither of them can reproduce the complexity of human periodontal tissues, making the development of a successful 3D oral mucosal model a necessity. The soft-tissue attachment formed around a tooth or an implant function like a biologic seal, protecting the deeper tissues from bacterial infection. The aim of this review is to explore the advancements made so far in the biofabrication of a junctional epithelium around a tooth-like or an implant insert in vitro. This review focuses on the origin of cells and the variety of extracellular components and biomaterials that have been used for the biofabrication of 3D oral mucosa models. The existing 3D models recapitulate soft-tissue attachment around implant abutments and hydroxyapatite discs. Hereby, the qualitative and quantitative assessments performed for evidencing the soft-tissue attachment are critically reviewed. In perspective, the design of sophisticated 3D models should work together for oral immunology and microbiology biofilms to accurately reproduce periodontal and peri-implant diseases.

## 1. Introduction

In the 1900s, “the gingiva” appeared to be the “apple of discord” in the field of periodontology, as it has been the subject of study and has generated in-depth discussions on the nature of periodontal soft-tissue attachment [1]. According to Gottlieb [2], the gingiva is organically united to the tooth surface, whereas Waerhaug [3] supported the idea that the epithelial cells adhere weakly to the tooth. Currently, it is widely acknowledged that gingiva functions as a barrier, as it attaches to the root surface, and it protects the underlying tissues of the periodontium from bacterial stimuli and other intraoral challenges. Longitudinal studies in the natural history of periodontitis suggest that this barrier of soft tissues may not be resilient to subgingival plaque accumulations, which may lead to tissue inflammation and destruction [4] and ultimately the development of periodontitis in susceptible individuals [5].

In recent years, periodontitis has been considered a chronic multifactorial inflammatory disease of the periodontium associated with subgingival biofilms in a predisposed host [6]. Periodontitis destroys the connective tissue and crestal bone, resulting in apical migration of the junctional epithelium (JE) and the formation of a periodontal pocket with inevitable tooth loss if left untreated [7]. It is the sixth most common chronic infectious non-communicable disease in humans [8], which undoubtedly affects patients’ general health, facial aesthetics, and quality of life [6]. According to the Global Burden of Disease study (2016), the prevalence of severe periodontitis is estimated at 11% worldwide [9]. Recently, a white paper by the Economist Intelligence Unit (EIU) highlighted that the prevention and management of periodontal diseases would help countries save considerable socio-economic costs over the following 10 years reporting from EUR 36 billion in Italy to EUR 7.8 billion in the Netherlands [10].

The gingiva is the part of the oral mucosa that covers the alveolar processes and surrounds the cervical region of the teeth. The oral epithelium, which lines the outer surface of the gingivae, transitions into the sulcular epithelium within the gingival sulcus interfacing the tooth, and then into the junctional epithelium (JE), which attaches to the tooth. The JE is the first line of innate host defence in the periodontal tissues [11]. The understanding of the complex mechanisms that occur within the periodontium during the development of periodontitis, and especially within the JE, will lead to justified clinical decisions and to the design of effective treatment strategies. Over the years, animal experiments and in vitro two-dimensional (2D) models have tried to study and better understand the host–bacterial interactions in the gingival sulcus. Animal experiments have been used in periodontal research and have largely contributed to unravel different biological aspects of inflammation and periodontal wound healing [12]. Due to innate differences between the animal species and humans, any direct comparisons between the two may be misleading or inconclusive. In addition, animal testing is costly and raises ethical concerns, as more than 12 million animals per year, including mice, monkeys, dogs, etc., are used in research units in Europe [13,14]. One of the most important limitations in animal models is the low predictive validity in drug testing, as 9 out of 10 drugs that pass animal testing fail in human clinical trials [15]. However, animal experiments still comprise a reliable research model, as they can be designed specifically to address a given research hypothesis [12].

In vitro study models and conventional 2D culture models used in basic cell biology offer a simple setup and an easy observation of cellular events. Nevertheless, their lack of mimicking native tissue in three-dimensional (3D) spatial order involving complex interactions between microorganisms and resident cells makes the development of more sophisticated in vitro tissue platforms a necessity [12]. To this end, 3D culture techniques are currently developed and aim to recapitulate the in vivo architecture and microenvironment of native tissues [16]. Bioengineered tissue models can reduce dependency on animal testing; develop reliable drug testing and improve toxicity testing for compounds and agents used in periodontal treatment; expedite access to new treatments; and develop personalised medicine by replicating an individual’s specific physiology, elements of the host response, and the subgingival microbiota. This can lead to cost-effective treatment interventions. The 3D tissue models can also enhance education and training by offering students realistic and interactive models of the periodontal apparatus. However, even the most sophisticated 3D gingival model cannot reproduce all the features of a living organ. For example, there is no gingival model successful enough to reconstruct the epithelium and the vascular endothelium interface [17,18]. So far, there is no clear standardised protocol to reproduce a 3D model of periodontal tissue attachment to a tooth surface, as it is in the oral cavity.

The purpose of this review was to comprehensively discuss novelties in the in vitro construction of the human gingiva and JE, focusing on technological advancements in the design of oral soft-tissue models, their challenges and limitations, and to address future directions in research.

## 2. Anatomy of the Human Gingiva/Mucosa

**Figure 1:**
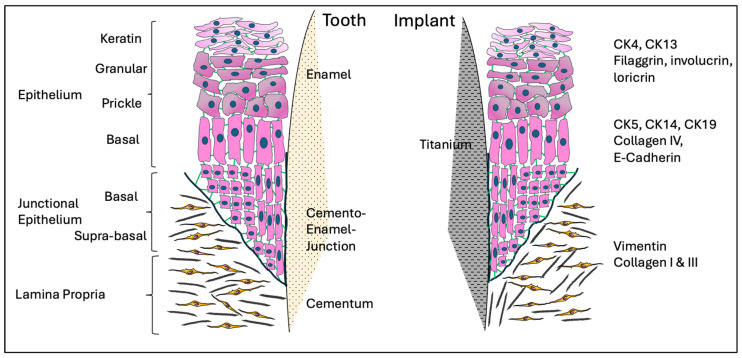
Schematic representation of the structure of the junctional epithelium in contact with the cemento–enamel junction of a tooth or with an abutment of a titanium dental implant. The epithelium is composed of dense layers of keratinocytes (pink cells) that have tight cell–cell junctions through E-cadherins; epithelial cells attach to the enamel or titanium with hemi-desmosomes (green lines). The conjunctive tissue (lamina propria) is mainly composed of gingival fibroblasts (yellow cells) embedded in the extracellular matrix rich in collagen type-I and type-III (red/green springs), elastin, fibronectin, laminin, and hyaluronic acid. Markers of the different layers are indicated on the right.

**Figure 2:**
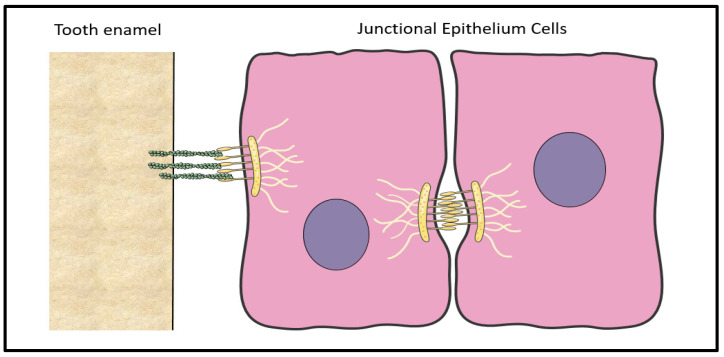
Schematic representation of the junctional epithelium at the cemento–enamel junction. The junctional epithelium cells maintain epithelial integrity by binding to the laminin fibres (green) on the tooth enamel by hemi-desmosomes (yellow) and by forming cell–cell tight junctions.

The gingiva consists of three types of epithelia based on histology, namely the oral, the sulcular, and the junctional epithelium, as illustrated in Figure 1. The oral epithelium covers the outer surface of the gingiva, while the sulcular epithelium lines the inner part of gingival tissue facing the tooth. Apically, it is continued by the junctional epithelium (JE), which is attached to the tooth surface and forms the base of the gingival sulcus. The oral epithelium is a keratinised stratified squamous epithelium and consists of the basal, prickle, granular, and keratinised cell layers. On the contrary, the sulcular epithelium is a non-keratinised stratified squamous lacking the keratinised cell layer. The JE is a key component of the gingiva, as it forms the epithelial attachment to the tooth [1]. Its detection and study has been strongly related to Schroeder H.E. and Listgarten M.A. (1971), who demonstrated that the JE is a stratified squamous non-keratinized epithelium that is tightly attached to the tooth via hemi-desmosomes [19,20]. The JE forms a collar-like band around the cervix of the tooth and its length ranges from 0.25 to 1.35 mm. It becomes narrower at the end, having a tapered shape, as it is formed by 15–30 cell layers coronally and by only 1–3 cell layers apically [21]. It consists of two layers, the basal and the supra-basal layers. The basal cells are adjacent to the underlying connective tissue via the basement membrane. The coronal supra-basal cells are in continuity with the sulcular epithelium and face the gingival sulcus, whereas the innermost supra-basal cells, which are called “Directly Attached to the Tooth (DAT)” cells, produce the internal basal lamina, which face and bind onto the cementoenamel junction [22]. The basal cells from all three epithelia are cuboidal, whereas the supra-basal cells at the JE are flattened with their long axis parallel to the tooth surface [23]. The development of the JE takes place during tooth eruption. Specifically, cells derived from the reduced enamel epithelium go through a slow rate transformation to JE after or shortly before their contact with the oral gingival epithelium [19,20,24]. However, the epithelial attachment can be re-formed de novo, even without the presence of a reduced enamel epithelium [24]. In detail, Listgarten et al., (1967) demonstrated that the gingiva was able to regenerate and re-attach to the tooth by hemi-desmosomes following gingivectomy [25]. There is evidence that the healing process of a periodontal defect following periodontal therapy may lead to periodontal repair with the formation of a long junctional epithelium attached on the root surface [26,27]. The long junctional epithelium is a continuity of the oral epithelium that might also contain remnants of the JE and the epithelial cell rests of Malassez (ERM), which originate from the Hertwig’s epithelial root sheath. During healing, epithelial cells form a non-keratinized epithelium that firmly attaches to the tooth by expressing adhesive proteins (laminin-γ2, integrin-β4, and -α3) similar to those secreted by the JE [28]. However, the long JE has a slower turnover than the JE [29] and is associated with a less efficient form of soft-tissue attachment against bacterial invasion [30]. Despite this, its formation post-therapy is not of clinical concern, as histological analysis indicates that the long junctional epithelium provides an efficient barrier function similar to that of the JE against microbial plaque accumulations [31]. In addition, the high turnover rate of the JE offers an important advantage, as its constant renewal protects the underlying tissues [1]. The cells of the JE are interconnected by desmosomes, gap junctions, and tight junctions [21,32,33], which form wide intracellular spaces, as illustrated in Figure 2. These spaces are filled with fluid, which derives from the subepithelial blood vessels found in the connective tissue. Of importance, the JE cells play a major role in innate immune responses by expressing various antimicrobial molecules such as interleukin-8, interleukin-1α, tumour necrosis factor-α, and matrix metalloproteinase-7. Langerhans cells and various types of defence cells such as neutrophils, macrophages, plasma cells, and phagocytes are present close to the basal layer of the epithelium and play an important role in health and disease [21,34]. Of note, polymorphonuclear leukocytes (PMNs) are found among the epithelial cells [19], with approximately 30,000 PMNs per minute migrating from the epithelium into the gingival sulcus, even under conditions free of clinical signs of inflammation [24,35]. The PMNs produce antimicrobial peptides and proteins, such as α-defensins, which contribute to the innate host defence [36]. Also, abundant lysosomal bodies and antibodies are found in the JE and participate in the host defence against bacteria. This remarkable cellular and molecular diversity within the gingival sulcus and the adjacent tissues safeguards periodontal health and highlights the significance of the JE in the etiopathology of periodontal diseases.

An extracellular matrix (ECM) is found between the basal cells of the JE and the gingival connective tissue (Figure 1). It is referred to as the external basal lamina or more simply as the basement membrane [21]. It functions as a selective barrier and it participates in cell polarisation and differentiation [21]. The external basal lamina consists of collagen (IV and VII), laminin, proteoglycans (heparan sulphate proteoglycan), and glycoproteins (fibronectin), while the internal basal lamina lacks collagen types IV and VII and most laminin isoforms [21,37,38,39].

The lamina propria is primarily composed of fibroblasts, vessels, and nerves integrated within the ECM. Fibroblasts, the predominant cell types of the gingival connective tissue, produce the ECM, which contains collagen fibres (type-I and type-III), elastin, laminin, fibronectin, and other proteins essential for the normal function of the connective tissue. In addition to fibroblasts, mast cells, macrophages, and other inflammatory cells contribute to tissue homeostasis and participate in the defence against oral microorganisms [40]. The gingival vascular system originates from the supra-periosteal blood vessels and the vascular plexus of the periodontal ligament. Beneath the JE, the supra-periosteal blood vessels form the dentogingival plexus, which lacks any capillary loops in a healthy state [23]. These vascular sources supply gingival tissues with oxygen, nutrients, host defence cells for an effective immune response, and platelets and growth factors necessary for wound healing.

At dental implants, the mucosa is attached around the metallic surface of an implant fixture—most commonly made of titanium—to form a soft-tissue barrier that protects the deeper tissues from challenges of the oral cavity [23]. As shown in Figure 1, this attachment is established either following implant installation (one-stage surgery) or following abutment connection (two-stage surgery) without having any histomorphometrical and anatomical differences. The peri-implant mucosa is 3–4 mm long when measured from the mucosal margin to the bone crest on the buccal aspect [41]. The peri-implant mucosa is characterised by three types of epithelia, which originate from the oral epithelium [42]. When keratinised, the outer surface of peri-implant mucosa consists of keratinised oral epithelium similar to the gingivae. The inner part, which faces the implant, contains the sulcular epithelium and an apical thin barrier epithelium similar to the JE [43].

Epithelial cells of the barrier form a thin layer of 3–4 cells and adhere around the implant surface via the internal basal lamina and hemi-desmosomes [44,45,46]. Animal and human histological studies have shown that the length of the barrier epithelium terminates approximately 2 mm apical to the most coronal peri-implant mucosa margin. An experimental study in rats compared histologically the JE around teeth with the peri-implant barrier epithelium. This study demonstrated that JE homeostasis is supported by a stem cell population with a high turnover rate, which was not observed in the barrier epithelium. Of interest, this soft-tissue barrier was characterised as chronically inflamed tissue due to fibrosis observed in the underlying connective tissue [47]. Regarding the connective tissue around dental implants, the collagen fibres which originate from the periosteum of the bone crest are parallel to the implant surface of approximately 1.0–1.5 mm long [48]. Of note, the peri-implant mucosa contains more collagen and fewer fibroblasts compared to the gingival tissue. Another difference in the two tissues is the vascular supply of the peri-implant mucosa, which originates only from the supra-periosteal blood vessels. Only few vessels are found in the peri-implant connective tissue forming a vascular plexus lateral to the barrier epithelium [49]. All these differences in the composition and anatomy of the soft tissues may in part explain different responses noted in peri-implant versus periodontal tissues to biofilm accumulations [50].

## 3. Origin of Cells Used in 3D Culture Gingiva Models

Cells of various origins have been used in the development of in vitro gingival models including primary cells of human or animal origin, commercially available cell lines, mesenchymal stem cells isolated from various intraoral sources, and induced pluripotent stem cells (iPS). Each cell type has its own advantages and inconveniences, and the choice depends on the study’s design and the researchers’ expertise [51,52].

Organotypic culture models have been developed in academic research laboratories, and they are now commercially available. MatTek Corporation, for instance, utilises oral keratinocytes harvested from either buccal mucosa or the gingival epithelium of healthy donors and produces non-keratinized (EpiOral) or keratinized (EpiGingival) stratified epithelium tissue [53]. These cells are cultured on a microporous membrane under serum-free conditions. Moreover, a full-thickness gingival model, including the lamina propria (EpiGinival-FT), is commercially available, which contains gingival fibroblasts in a collagen matrix harvested from healthy donors [54,55]. In addition to MatTek, EPISKIN is a French enterprise that employs cultures of TR146 cells derived from a squamous cell carcinoma of the buccal mucosa on an inert polycarbonate filter at the air–liquid interface, producing the SkinEthic-reconstructed Human Oral Epithelium (HOE) [56]. The same company is producing the SkinEthic Human Gingival Epithelium by culturing normal human gingival cells (non-cancerous keratinocytes). Commercially available cell lines provide an ethical alternative to animal experiments and can be useful as they offer an easily accessible source, can provide reproducible and reliable results for in vitro experiments, and can have an infinite proliferation capacity [57]. Moreover, as they are ready to use, the timeframe of an in vitro experiment is expedited [53]. However, evaluations of their authenticity and how accurately they represent primary cells should be frequently conducted, and findings should be interpreted with caution, as their responses may differ from those of primary gingival cells [58].

Primary cells are isolated from gingival biopsies of human or animal origin. Primary epithelial tongue cells, for example, originating from mice were embedded in hydrogel enriched with growth factors, i.e., epidermal growth factor, R-spondin, and fibroblast growth factor, and they successfully formed oral mucosa organoids [59]. However, animal-derived primary cells share the same limitations with animal experiments, making them unsuitable for human-oriented 3D experiments. Human-derived primary cells are a valuable source for patient-oriented treatment but caution should be taken due to any biological variability that may occur during the donor’s life and across different donors [60]. Moreover, primary cell cultures are limited by their short lifespan, as they cannot proliferate indefinitely [61]. To address this shortcoming, it has been found that the introduction of human telomerase reverse transcriptase (hTERT) immortalises cells, retaining their parental cell characteristics [62]. Bao et al. used human papillomavirus oncoproteins (E6, E7) to immortalise gingival keratinocytes and fibroblasts so that they could be expanded for more than 30 passages. These immortalised cell cultures were then used to fabricate in vitro a standardised organotypic gingival model [63].

Induced pluripotent stem (iPS) cells derived directly from somatic cells remain a promising method for personalised regenerative medicine [64]. More specifically, these stem cells are named pluripotent because they can differentiate into any cell type, including neurons, endothelial cells, osteoblasts, and more [65,66,67]. In 2006, Takahashi and Yamanaka successfully reprogrammed mouse dermal fibroblasts to iPS cells by using retroviral gene transfer with four transcription factors, Oct3/4, Sox2, Klf4, and c-Myc [64]. In dental research, human gingival fibroblasts have been successfully generated into iPS cells by introducing a retroviral transduction cocktail of OCT3/4, SOX2, KLF4, and c-MYC [68]. The iPS cells efficiently expressed embryonic stem cell markers such as SSEA4 and OCT4. However, the cells induced teratoma formation after their implantation into murine models. Besides tumorigenicity, iPSCs may present poor differentiation quality and a low growth rate due to an incomplete induction of pluripotency [69,70]. For that reason, achieving successful reprogrammed iPSC lines is challenging and all steps during the reprogramming process should be optimised and standardised, addressing all possible risks for clinical application in humans.

## 4. Biomaterials and ECM Derivatives Employed in 3D Culture Models

Multiple cell types used in 3D oral mucosa cultures interact not only between them but also with the ECM components creating tissue-like structures [71]. For the biofabrication of the oral mucosa, scaffolds are employed to recapitulate the ECM component of native oral soft tissues and to provide “closer to in vivo” cell behaviours such as cell adhesion, proliferation, and differentiation. An ideal scaffolding material should be biocompatible, i.e., should not cause any harm, be biostable, i.e., should resist microorganism effects, and have a high level of mechanical properties, avoiding tissue collapse during its manipulation. Natural or synthetic scaffolds have been widely used in previous 3D culture experiments. Natural scaffolds of collagen in various forms (i.e., sponges, hydrogels, membranes, etc.) can be derived either from animals such as rat, pork, or bovine cells or from humans, i.e., de-epithelialized cadaver dermis or human amniotic membranes [72,73,74,75,76]. The most commonly used matrix is collagen type-I from the rat tail tendon that mimics the human ECM and supports the stratification of the epithelial layer and homogenous distribution of fibroblasts [73,77]. The drawback of the models employing collagen type-I is that they demonstrate shrinkage in culture [78]. Additionally, the heterogeneity of commercially available batches of collagen, the cost of the material, and the risk of immunogenicity necessitate the use of synthetic or recombinant collagen alternatives of human or animal origin [79]. Collagen sponges and gels consisting of collagen type-I and type-II have also been tested for their suitability as a matrix in cell culture. Collagen gels are characterised by marked shrinkage during fibroblast incorporation and culture, leading to collagen synthesis inhibition from fibroblasts [80,81]. In contrast to collagen gels, collagen sponges permit cell proliferation within their porous structure [82]. It has been observed that gingival fibroblasts can successfully attach to collagen sponges and keep their cell metabolic activity stable throughout the experiment [83]. Nevertheless, collagen sponges also tend to swell or contract during culture, which may influence its properties. In addition, their high opacity complicates cell microscopy, as it impedes the visual examination of the internal structure [81].

A promising alternative to 3D experiments is the dermal substrate [73]. In a 3D mucosa model, the epithelial layer presented a higher proliferation, stratification, and differentiation potential when co-cultured with a human-sourced acellular dermal matrix (Alloderm) compared to a rat collagen matrix [74]. Nevertheless, acellular dermal matrices may hinder fibroblast cells in growth, as they proliferate only at the outer surface of the matrices [84]. In another experiment by the same research group, a porcine acellular dermal matrix (Strattice) was used to create an ex vivo-produced oral mucosa-equivalent model and to assess molecular changes in keratinocytes when challenged by phototherapy [85]. However, dermal matrices are more expensive than synthetic materials and have limited availability [73].

Other innovative approaches include a micro-patterned fish scale collagen type-I scaffold with chemical cross-linking, which was constructed using a micro-electromechanical systems process and soft lithography [86]. The scaffolds have viscoelastic properties and allow for the development of epithelial ridge-like structures, known as rete ridges, that enhance the connection between the epithelium and the connective tissue by increasing the length of the interface between the two tissues [87].

A silk protein porous scaffold has also been used to reproduce host–pathogen interactions which take part in the periodontal pocket [88]. The silk proteins, derived from silkworm cocoons, were engineered into biopolymers by casting a replica mould of a human mandible, where human primary gingival cells were seeded to recreate the dentogingival junction and support oxygen and nutrient diffusion. This scaffold represented a functional gingival construct, where shifts in inflammatory markers were detected when an oral microbiome was inoculated in the model [89]. The batch variability due to different types of silkworms, the high sensitivity of processing techniques, and the contamination potential were significant drawbacks of this approach.

Synthetic scaffolds such as Vicryl, polyglycolic acid (PGA), or polylactic-co-glycolic acid (PGLA) do not comprise the first choice for 3D oral mucosa culture models based on the literature. A tissue-engineered human oral mucosa model was fabricated by culturing fibroblasts in Vicryl scaffolds [90]. Vicryl is a surgical mesh of woven polyglycin 910, a copolymer of glycolide and lactide in the form of a membrane. This model showed that when synthetic material was used for the ECM, primary oral keratinocytes and fibroblasts failed to grow in stratified layers and instead proliferated in the periphery of the Vicryl membrane where they were seeded. In another study, polyvinyl alcohol and gelatine from bovine skin were used to create a spongy scaffold aiming to observe the morpho-functional behaviour of growing human gingival fibroblasts in a 3D culture model [91]. Fibroblasts adhered successfully to the scaffold and produced ECM components like collagen type-I, fibronectin, and laminin. Biocompatibility issues of alloplastic materials limit their use in 3D human cultures. To date, no biomaterial substrate has demonstrated clear superiority in recapitulating a native ECM, making it challenging to accurately engineer a 3D gingival model that truly mimics nature.

## 5. Reconstruction and Characterisation of the JE in In Vitro 3D Oral Mucosa Models

Numerous attempts have been made to reconstruct a 3D gingival model which is reproducible and mimics the architecture of the native tissues, including the JE formation around an insert such as an implant or a tooth. As reported in Figure 1, the reconstructed gingival tissue should express specific biomarkers evidenced by immunofluorescence confocal microscopy, histology, or RTqPCR that are normally observed in native tissues.

Such 3D tissue models need advanced imaging methods for analysis, which provide critical insight into the structure, function, and biochemical properties. Main techniques include confocal microscopy, in which a laser beam provides high-resolution optical sectioning, which is considered excellent for a detailed visualisation of cells and the distribution of biomolecules and two-photon microscopy enabling deep tissue imaging with minimal photodamage, thus being appropriate for live tissue observation with regard to dynamic cellular processes [92]. Light sheet fluorescence microscopy limits photodamage by illuminating the sample from multiple angles with a ray of light and is well suited for large specimens, such as organoids [93]. Furthermore, micro-computed tomography offers high-resolution 3D imaging of internal structures and is one of the most common techniques to evaluate the microarchitecture of the engineered tissues [94]. An alternative assessment method is fluorescence lifetime imaging microscopy, which by measuring the fluorescence decay investigates molecular interactions and cellular metabolism. Furthermore, scanning electron microscopy provides high-resolution images of the sample surfaces for morphological analysis in tissue constructs. Of note, the in vivo imaging techniques allow the monitoring of engineered tissues in living organisms, enabling functionality and integration assessment. All together, these capabilities allow for taking a better look at an otherwise complex biological process while improving the design and functionality of the engineered tissues for major applications in regenerative medicine and drug discovery.

Initially, partial thickness 3D platforms were developed to recapitulate either a fully differentiated stratified epithelial layer or a connective tissue layer [85,95]. Furthermore, various full-thickness gingival models exist in which epithelial cells are co-cultured with gingival fibroblasts in a collagen matrix and they form an epithelial layer and an underlying connective tissue layer, respectively [96]. However, few gingival models focus on the construction of the JE. There are still no clear criteria to identify the JE cells in a 3D oral mucosal model. In an innovative study for fabricating the oral mucosa, immortalised primary human gingival keratinocytes (HGEK-16) and fibroblasts (GFB-16) were selected and co-cultured in a 3D collagen matrix originating from rat tail collagen type-I [63]. The HGEK-16 cells formed a multilayered epithelium similar to the JE, as the keratinized layer was not observed, and qRT-PCR confirmed the expression of cytokeratins CK-10, -13, -16, -18, and -19 within the epithelium. The quantitative and qualitative analysis indicated that this 3D organotypic system is a step closer to human gingival tissue compared to conventional monolayer cultures. The main advantage of this platform is reproducibility, although it lacks the expression of JE-specific markers due to the absence of a tooth or an implant insert [63].

In another study, the JE characterisation was further investigated using specific markers, while the influence that the culture time had on the gingival epithelium phenotype was also studied. Indeed, gingival keratinocytes, gingival fibroblasts (HGFs), and periodontal ligament fibroblasts (HPLFs) were isolated from the extracted wisdom teeth of 12 healthy individuals [96]. Epithelial cells were seeded on top of a collagen substrate (origin of which is not reported) that contained either HGFs or HPLFs, and each model was lifted at the liquid–air interface to achieve epithelial stratification. Interestingly, on the fifth day of culture, the epithelium which was formed above the connective tissue-like layer of the HPLFs closely resembled the JE, as it lacked keratinisation and had fewer cell layers (3–5 layers) compared to later stages of culture (9–13 layers on the seventh day of culture). Specific markers characteristic for the JE were expressed within this construct, like Ki-67, a marker of cell proliferation, odontogenic ameloblast-associated (ODAM) protein, follicular dendritic cell-secreted (FDC-SP) protein, and CK-8, -13, -16, and -19. The ODAM protein is used to characterise the JE without fully understanding its molecular function [97]. The FDC-SP protein seems to play a role in host defence [98], while the CK-19 protein is a specific marker constantly expressed by the JE cells [99]. On the ninth day of culture, a higher differentiation potential was observed in models cultured with HGFs and the expression of CK-19 was reduced significantly, suggesting a resemblance to the oral gingival epithelium. This model demonstrated that culture time and the type of fibroblasts may affect the epithelial phenotype. However, in that study, the characterisation of epithelial cells was performed based on the expression of molecular markers without providing further information of the shape and spatial organisation of the JE-like cells.

A more recent study took a step further by investigating the effects of different fibroblast populations on the development of the oral gingival epithelium and the JE using an organotypic in vitro model [100]. During tooth extraction, samples of the tooth with the attached gingival tissue were harvested and proceeded with the micro-dissection of the gingival tissue, cell isolation, and finally, cell amplification in a 2D culture. Human gingival fibroblast or HPLF cells were embedded in a collagen gel originating from rat tail tendons. Cells isolated from the oral or the junctional epithelium biopsies were seeded on top of the connective tissue analogue and cultured at the air/liquid interface for 14 days. Specific markers such as CK-19, glycoprotein dolichos biflorus agglutinin (DBA), and protease matrix metalloproteinase (MMP)-7 were used to characterise the JE, while the CK-10, -4, and -13 markers characterised the oral gingival epithelium. The seeded JE cells formed a single layer when cultured with the HPLF but showed increased thickness when cultured with the HGFs. This study sheds light onto the role that fibroblasts play in the growth of epithelial cells without unveiling the mechanisms behind these interactions. A similar model was developed by using H400 cells, an immortalised human cell line derived from precancerous oral carcinoma [101]. The H400 cells were cultured on top of a bilayer connective tissue analogue comprising HGF cells on top and HPLF cells on the bottom of a recipient collagen bed of uneven dimensions. The HPLF layer had a greater horizontal outgrowth than the overlying HGF layer [102,103]. It was suggested that the proliferation and migration of the H400 epithelial cells were hindered following contact in culture with the HPLF cells, implying that the HPLF cells regulate the growth of the epithelium. This study demonstrated that the HPLF cells strongly expressed the secreted frizzled-related protein-4 (SFRP4). The SFRP4 is responsible for the downregulation of the Wnt signalling cascade, which plays an important role in tissue homeostasis [104]. Therefore, the inhibition of epithelial downgrowth may be caused due to the expression and diffusion of the inhibitor SFRP4. The studies mentioned above point out that the origin of fibroblasts may influence the morphology, architecture, and growth of keratinocytes during culture.

Interestingly, an attempt has been made in the literature to incorporate vascularity within the 3D-constructed tissues. It has been shown that the co-culture of human periodontal ligament-derived stem cells (PDLSC) and endothelial cells enhances the formation and the number of blood vessels in vitro [105]. A hypothesis has been recently tested whether gingival fibroblasts also have an angiogenesis potential when they are co-cultured with human umbilical vein endothelial cells (HUVECs) [106]. For this, a 3D culture model was developed in a composite matrix of collagen hydrogel of type-I collagen rat tail origin or of methacrylated collagen and hyaluronic acid. The findings highlighted the potential of the HGF cells to express perivascular markers and support a long-lasting HUVEC network. However, the role of HGFs in the vascularisation of 3D gingival culture models requires further study.

## 6. Three-Dimensional Culture Models Employing Implant Abutment Units

Moving forward in the complexity of the reconstructed tissues, efforts have been made to recapitulate the implant soft-tissue interface [75]. A series of studies from the same research group co-cultured TR146 oral keratinocytes and HGF cells onto a human-origin acellular dermis membrane (Alloderm), which was completely submerged in the medium to fabricate an in vitro 3D oral mucosal model [75]. On the fourth day of culture, a 4 mm hole punch was created by a disposable tissue punch (Stiefel Laboratoire, UK) to accommodate a titanium (Ti) disc of 5 mm diameter and cell culture was continued for 10 days. Different Ti types were used in terms of surface roughness (polished or machined) and surface treatment (sandblasted or anodized). None of the titanium inserts showed significant differences in the permeability of the mucosa analogue attachment. Also, a few hemidesmosome-like structures were detected with transmission electron microscopy (TEM) to evidence soft-tissue attachment upon the titanium surface.

The assessment of soft-tissue attachment onto an implant abutment of 2.5 mm collar height was also investigated after constructing a 3D mucosal model. A fully stratified epithelial layer of immortalised human keratinocytes (KC-TERT, OKG4/bm1/TERT) was seeded over a 3D layer of immortalised gingival fibroblast cells (Fib-TERT) grown in collagen hydrogel of rat tail origin [107]. After 10 days of culture, the abutments were inserted into the modelled tissues after creating a 3 mm hole punch and the models were incubated for 10 more days. Then, the epithelium was dissected carefully from the implant unit and was fixed with formaldehyde. Histomorphometric and immunohistochemistry analyses of the tissue analogue suggested the fabrication of a stratified epithelium, where CK-4 and Ki-67 were expressed in the upper cell layers and CK-19 was only expressed in the basal cell layer at the interface with the abutment. In addition, laminin-5 and collagen-IV were produced within the lamina propria, indicating the formation of a basement membrane.

Furthermore, the formation of soft-tissue attachment has been investigated on surfaces of different implant posts of 5 mm diameter and 3 mm height, i.e., sandblasted acid-etched (SLA), machined Ti, titanium nitride-coated (TiN-coated), zirconia, and polyetheretherketone (PEEK) surfaces [54]. Histology and scanning electron microscopy (SEM) were utilised to assess the outcomes of the experiment. The 3D model used was fabricated by seeding primary human gingival epithelial cells over a connective tissue analogue of HGF cells cultured in an electrospun bovine collagen-I gel matrix. At day 7, the implant posts were placed in the middle of each culture tissue analogue using a 5 mm sterile biopsy punch and the inserts were left in situ until day 14. According to a histomorphological analysis, a stratified keratinised epithelium was observed. Nevertheless, it was a third of the thickness of the human native gingiva lacking rete peg formation. A variability in the soft-tissue attachment onto the various posts was shown by histology. PEEK and Ti machined surfaces showed an attachment from both epithelial and connective tissue, whereas SLA and zirconia surfaces primarily exhibited sole epithelial attachment. No tissue attachment was observed on the TiN surface.

In another study, Barker et al. fabricated a similar 3D model and they suggested higher cell viability when implant inserts of an SLA surface were used compared to zirconia, ceramic, or PEEK surfaces [108]. In that study, regardless of the implant surface, epithelial adhesion on all implant inserts was demonstrated by SEM. Peri-implant mucosal attachment to zirconia abutments is also a point of interest. Human primary gingival epithelial cells and fibroblasts were co-cultured onto a round acellular human cadaveric dermis membrane (Alloderm). In the centre of the membrane, a punch hole was created by a disposable tissue biopsy punch and the zirconia implant analogues were inserted. The cultures were incubated for 10 days. Histological analysis showed the formation of a stratified squamous epithelium and the biological seal of the epithelial attachment onto the insert was assessed by the volume of radioactive water penetrating this seal. Limitations of this model include the lack of a well-structured gingival model and inadequate characterisation of the soft-tissue adhesion [76].

Attempts have been made to incorporate pathogenic bacteria into the 3D culture models to assess the impact they have on soft-tissue attachment. In a peri-implant 3D model, a Ti disc was integrated into a 3D culture analogue of HGFs cultured in collagen gel of rat tail origin. Oral immortalised keratinocytes (OKF6/TERT-2) were seeded on top of the collagen–fibroblast substrate and the model was cultured for 25 days [109]. Over the last 24 h of the experiment, the peri-implant mucosa was challenged with either *Streptococcus Oralis* or *Aggregatibacter actinomycetemcomitans*. The bacteria were added onto spacers located on top of the Ti disc, facing the mucosa. Immunohistochemistry revealed a slight loosening of the epithelium facing the *S. oralis* biofilm. Furthermore, a downregulation of the inflammatory response was observed in the models infected by *A. actinomycetemcomitans*, whereas a balanced immune response was present when *S. oralis* was inoculated into the mucosa model. These data demonstrate that host responses in relation to native tissue take place in the reconstructed gingival tissues.

## 7. Methods of Qualitative and Quantitative Evaluation of the Soft-Tissue Attachment

There is ample evidence in the literature that verifies the soft-tissue attachment on implant units through quantitative assessments, such as basic histology using staining [75,76], permeability tests, attachment tests [75], and qualitative assessments, including light microscopy after ground sectioning [110], transmission electron microscopy (TEM) [111], scanning electron microscopy (SEM) [54,75,107,108], and lastly, the measurement of trans-epithelial electrical resistance (TEER) [112].

Histological analysis under light microscopy has confirmed the parallel growth of epithelial cells to a zirconia implant abutment [76]. Barrier properties of the soft-tissue analogue have been studied through permeability tests, which included measurements of the amount of a radioactive substance penetrating the mucosal-modelled attachment. Chai et al. (2012) suggested that experimental groups with the presence of an epithelium attachment had a lower permeability compared to those that lacked an attachment [75]. In addition, an attachment test was performed, where the insert was pulled out gently from the tissue analogue and an Alamar Blue assay was used to measure the viability of cells that remained attached onto the implant insert. An attachment was observed in different abutment topographies, implying that the insert topography does not play a significant role in the cell-to-insert attachment.

The qualitative assessment of the soft-tissue attachment on an implant insert necessitates the preservation of this interface during specimen preparation [113]. The specimen preparation is challenging and can be realised by histological sections either by maintaining the insert, as in ground sectioning [114,115], or by employing the “cryofracture procedure” [116] or electropolishing techniques [110,117] after removing the insert. The ground sectioning technique can preserve the direct interface of the soft tissue and the Ti surface. However, this technique is sensitive and there is a risk of damaging the sample during the procedure [110]. The cryofracture procedure involves freezing the specimen in liquid nitrogen and then immersing it in boiling water. This procedure leads to soft-tissue dissociation from the insert, but this technique has been assessed only in animal experiments not using in vitro 3D oral mucosa models [116]. The implant soft-tissue interface of specimens processed by ground sectioning or by semithin sectioning after electropolishing has been explored under light microscopy. Both techniques showed a peri-implant tissue, like the epithelium, attached to different Ti surfaces [118]. However, specimens following the electropolishing technique offered a more detailed assessment of the structure. Chai et al. proposed a new contour method analysis, which involved taking a silicone impression of the soft-tissue implant model and duplicating it into silicone polymer models [110]. Then, the models were sectioned with a scalpel blade into eight parts and these sites were examined under a stereoscope, observing the angle between the Ti disc and the fabricated tissue. While this technique may seem like a simple method for specimen preparation, it is highly sensitive and requires further research to optimise. Moreover, preparing ultrathin sections for TEM can be particularly difficult, especially when dealing with the hard surface of the implant. However, a focused ion beam (FIB) technique has been developed to provide highly detailed images by removing the bulk of the Ti, leaving only a thin layer despite the fact that artefacts may occur during the sample preparation [118]. Transmission electron microscopy analysis has further confirmed soft-tissue attachment through the observation of hemidesmosome-like structures at the interface of the soft tissue to the Ti discs [116]. In addition, cell morphology has been assessed on various abutment surfaces, showing that cells are flatter on machined titanium, zirconium, ceramic, and PEEK surfaces, while they appear more 3D when seeded on an SLA surface [108,111]. Of note, assessment by SEM has often been used for qualitative assessment and has confirmed cell attachment around the Ti surface regardless of the surface roughness [54,75,107,108].

A promising quantitative method for the evaluation of the cell barrier integrity is the measurement of trans-epithelial/trans-endothelial electrical resistance (TEER) [114]. This non-invasive method uses electrodes of various designs to detect changes in the tight junction proteins between cells. Tight junctions are cell intercellular junctions shaping a permeable barrier that regulates solute diffusion [21]. Riaz et al. fabricated a 3D oral mucosal equivalent to test its permeation barrier properties in comparison to a commercial model (EpiOral) [119]. A full-thickness model was fabricated by adding fibroblasts (NIH 3T3) on an acellular collagen gel derived from rat tails, and finally adding on top OKF6 TERT-2 keratinocyte cells. Using eletriptan hydrobromide as a model drug, TEER values were similar for the two models, indicating that the oral mucosa equivalent was a suitable model to study drug effects on cells. However, the TEER methodology is influenced by various parameters, such as porosity, model material, and the medium used for measurement. As a result, caution should be taken when comparing recordings from different culture models [118,120].

## 8. Organ-on-a-Chip Technology for Oral Mucosa 3D Models

Organ-on-a-chip technology has sought to develop advanced 3D culture models that closely mimic the complex environment and functions of oral tissues. These are microfluidic devices constituted by different layers of cells separated by a semipermeable membrane. In oral research, three primary chip designs are commonly used, namely one-chamber, multiarray, and parallel-chamber designs [112,121]. The one-chamber chip is the most common model, with only one culture chamber joined by channels for fluid transport. In contrast, the multiarray chip has multiple equal-volume chambers that are interconnected by channels and arranged in a matrix [112]. One of these models, the mucosa-on-a-chip, accurately simulates the oral mucosa, allowing for accurate monitoring of cellular responses to dental biomaterials and bacteria [122], also incorporating elements of the host response [123].

The mucosa-on-a-chip model is a sophisticated in vitro system to replicate the complex environment and functions of human oral mucosa. This model comprises a microfluidic device that creates a three-dimensional environment for the culture of oral epithelial cells. The chamber contains two parallel channels separated by a porous membrane enabling the co-cultures of two cellular types, such as epithelial and immune cells. The flow of culture medium through channels facilitates active nutrient exchange and waste elimination, effectively simulating physiological conditions in the oral cavity. This flow can be steady, where the flow rate does not change with time, or pulsatile, where it fluctuates to mimic the pumping of blood vessels [124]. Rahimi and his colleagues developed an oral mucosa-on-a-chip with a histologically based configuration of epithelial and fibrous layers. Fibroblasts were embedded in collagen in the central channel, and subsequently, keratinocytes were seeded into pores between polydimethylsiloxane (PDMS) posts on the apical layer. Their model has demonstrated sensitivity and efficiency in testing the cell responses against varied hydroxyethyl methacrylate concentrations and has allowed for an accurate tracking of cell responses to dental biomaterials and oral bacteria [112].

Likewise, the dentin-on-a-chip platform mimics the dentin–pulp complex, facilitating the study of the odontoblast processes by replicating the in vivo architecture of odontoblasts. In vivo odontoblasts are located at the periphery of the dental pulp, with cytoplasmic projections extending into the dentin tubules [125]. These projections are crucial for transmitting external stimuli. However, in traditional culture systems, this distinctive morphology is lost [126]. Niu et al. successfully recreated the dentinal architecture by using a dentin-on-a-chip model with two parallel chambers connected by multiple 2 μm microchannels, simulating the dentin tubules. Hydrostatic pressure was applied to guide odontoblasts from one chamber to the other. The narrow width of the microchannels prevented the entire odontoblast cell body from passing through, resulting in the formation of odontoblast projections [127]. Another dentin-on-a-chip model using odontogenic stem cells originating from the apical papilla (SCAP) further highlights the potential of this technology. By using gelatin methacrylate (GelMA) hydrogels at different concentrations, researchers were able to optimise the differentiation of SCAP cells at a GelMA concentration of 5% in a way that mimics the natural oral environment [128].

The first tooth-on-a-chip model consisted of two parallel channels, two perfusion chambers, and a central groove that hold a dental fragment. The fully assembled microdevice simulated the interface between dentin and dental pulp on one side and between dentin and dentin material on the other, creating two distinct chambers, namely the pulp side and the cavity side [129]. This model of the pulp–dentin–biomaterial interface was designed to study the interface between pulp cells, dentin, and various dental materials. It showed real-time responses of pulp cells to the test materials, comparing cytotoxicity and morphological changes to those observed in traditional in vitro models. Furthermore, a biomaterial–biofilm–dentin interface was established with *Streptococcus mutans*. Human dental pulp stem cells were cultured on dentin, creating a 3D extracellular matrix by incorporating collagen. This structure evaluated the antimicrobial properties of calcium silicate cement on the biofilm by demonstrating disruption of the structural integrity of the biofilm and death of the implicated bacteria [130].

Moreover, an epithelium–capillary interface-on-a-chip device has been developed to study inflammatory infiltrates within the bioengineered periodontal soft tissues. This device sequentially integrated HUVECs and human gingival epithelial cells (HGECs) to mimic the anatomy and microenvironment of periodontal soft tissues in vivo. Their results demonstrated that this innovative periodontal soft-tissue device was able to reproduce the inflammatory process induced by LPS or TNF-α in periodontal mucosa cell lines. In addition, it allowed for the measurement of multiple biomarkers in each cell line, enabling the study of the intercellular communication between them [131]. This in vitro epithelium–capillary interface microarray device appeared to have a potential to serve as a platform for studying drug-induced effects on the function of periodontal soft tissues in homeostasis and disease.

Models like the gingival crevice-on-a-chip study the interactions between host cells and oral bacteria in conditions of disease such as periodontitis [132], while the salivary gland-on-a-chip and oral cancer-on-a-chip examine salivary secretion and tumour development [133]. These advancements reduce the use of animal models and enable real-time analysis of cellular behaviour in a more precise biological environment. While there is still a number of technical challenges to overcome, organ-on-chip technology provides great promise for drug testing, tissue regeneration, and personalised therapies to improve oral healthcare.

## 9. Similarities and Differences Between the 3D Models and the Native Tissue

As reported in Table 1, there are specific characteristics of the oral epithelial tissues in contact with a tooth or a dental implant unit and the reconstructed 3D models aim to reproduce these features as closely as possible. It is widely acknowledged that 3D oral mucosal models are more clinically relevant to native tissues than 2D cell cultures [20]. It has been postulated that 3D tissue models show decreased variability compared to animal models [18]. Several methods have been developed and successfully used to construct a stratified squamous gingival epithelium that closely mimics the native tissue despite the fact that there is a great amount of variability in these protocols regarding the origin of cells, the matrix used, the technique for the 3D cell culture, etc. [20,22,23]. Across studies, immunohistochemistry analysis provides the evidence for the development of 3–6 epithelial cell layers in these 3D tissue analogues. According to Razali et al., the cultured epithelial cells were organised in a parallel direction with the abutment surface similarly to the native tissue [76]. Furthermore, intracellular connection via desmosomes and the connection of the modelled soft-tissue with the Ti surface via hemidesmosome-like structures have been observed in transmission electron microscopy (TEM) of epithelial layers [108]. The fibroblast cell layer has been shown to inhibit epithelial downgrowth along the surface of an implant abutment [76,100], and it can also influence the architecture of the constructed epithelium and the expression of markers.

Nevertheless, there are some differences in the existing 3D models compared to native tissues. Notably, there is lack of strong evidence on the proliferation, differentiation, and characteristics of cells in long-term culture conditions even after immortalised cells have been used. The quality of the soft-tissue attachment and its mechanical properties need further investigation, as there are only a few models in the literature so far that have reported on soft-tissue attachment onto implant abutment and tooth analogues [76,104,106,108]. The existing models simplified the replicated versions of the gingiva and the oral mucosa, as they lack vascularity, neurosis, and elements of an organised immune system, affecting the ability of the modelled tissues to grow and respond to different stimuli. Moreover, the rete pegs, which comprise a critical anatomic element of the oral epithelium due to their protective and absorptive properties, have not been modelled yet. The current static models do not enable dynamic flow, which supports nutrient and drug delivery and waste removal of the tissues [14]. The source of the materials and scaffolds used in 3D models varies and might be of animal, human, or synthetic origin, possibly having an impact on the cellular interactions and signalling pathways, which requires a careful interpretation of outcomes. Furthermore, the construction of a soft-tissue attachment onto an insert (implant or tooth) is often realised by Ti or hydroxyapatite discs, which have a different configuration than an actual implant abutment or a tooth insert, respectively.

The limitations mentioned above highlight the lack of established protocols for the biofabrication of a valid 3D gingival model. To address these challenges, at first, cell source quality and consistency should be performed in a consistent and robust manner. Experiment repetition by using multiple donors’ cells may lead to reproducible results and meaningful insights into a disease [14]. Confirmation by histology and molecular analyses of specific marker expression are ways to confirm the model’s validity. With the advancement of culture techniques and technologies, elements such as vascular, immune, neural, and bone cells will become feasible to incorporate in the 3D platforms to resemble the native tissue of a single species origin.

## 10. Conclusions

Three-dimensional human oral mucosa equivalents have been developed, aiming at studying the aetiopathogenesis and new treatments of periodontal and peri-implant diseases as an ethical alternative to animal experiments. However, the present oral models lack the incorporation of several components such as an osseous tissue equivalent, vascularisation, immune cells, and a biofilm component, which comprise important elements of the native tissue. Until today, 3D culture models have achieved the fabrication of a subunit of the oral mucosa but as there are still challenges to be addressed, the animal models are still useful and cannot be replaced yet. Future research could seek to construct 3D oral models that are inclusive of various elements regarding a tooth/implant insert embedded in a native-like multilayer soft-tissue analogue having vasculature, incorporating a microbial component and defence cells in long term culture conditions.

## Figures and Tables

**Table 1:** Characteristics of the oral epithelia and comparison with in vitro 3D models (n.a. non applicable; n.d. not determined).

Characteristics	Oral Epithelium	Junctional Epithelium	Peri-Implant Epithelium	In Vitro 3D Models
Type of epithelium	stratified, squamous, keratinized	stratified, squamous, non-keratinized	stratified, squamous, non-keratinized	
Cell layers	10–20 cells basal, granulosum, corneum	15–20 cells coronally, 1–3 cells apically (close to the tooth)	3–6 cell layers, thin	3–6 cell layers, thin
Length of epithelium	n.a.	0.25–1.35 mm	2 mm	n.d.
Keratinocyte cell shape	cuboidal (basal layer), irregular (prickle), flattened, and keratinized	cuboidal (basal layer), flattened (supra-basal)	n.d.	flattened cells in 3D
Intercellular connections	desmosomes, tight and gap junctions			desmosones
Soft-tissue tooth/implant attachment	n.a.	numerous hemi-desmosomes	few hemi-desmosomes	n.d.

## Data Availability

No new data were created or analysed in this study. Data sharing is not applicable to this article.
